# Lingering Impact of Starting Working Life During a Recession: Health Outcomes of Survivors of the “Employment Ice Age” (1993–2004) in Japan

**DOI:** 10.2188/jea.JE20190121

**Published:** 2020-09-05

**Authors:** Takashi Oshio

**Affiliations:** Institute of Economic Research, Hitotsubashi University, Tokyo, Japan

**Keywords:** age-period-cohort analysis, difference-in-differences analysis, employment ice age, self-rated health, subjective symptoms

## Abstract

**Background:**

A growing amount of evidence demonstrates the adverse impacts of economic downturns on population health. However, the extent to which the macroeconomic conditions at labor market entry affect health outcomes in later life remains relatively understudied. This study focused on the health outcomes of the cohort who entered the labor market during the “employment ice age” (EIA; 1993–2004) in Japan, when young people had difficulty finding jobs after graduating from college or high school.

**Methods:**

We used repeated cross-sectional data (*N* = 3,054,782; 1,500,618 men and 1,554,164 women) obtained from an 11-wave population-based nationwide survey conducted every 3 years from 1986 through 2016. We considered three health outcomes: being in hospital, subjective symptoms, and self-rated health (SRH). We employed two types of statistical analyses: an age-period-cohort (APC) analysis, which controlled for age and period (wave) effects, and a difference-in-differences (DiD) analysis, in which the EIA experience was regarded as a treatment.

**Results:**

The APC analysis confirmed the relative disadvantage of the EIA cohort for all three outcomes; for instance, the odds ratio of poor SRH for the EIA cohort was 1.29 (95% confidence interval [CI], 1.21–1.38) for men and 1.25 (95% CI, 1.17–1.34) for women. The DiD analysis confirmed the robustness of these results, especially for men.

**Conclusions:**

The results underscored the lingering impact of the macroeconomic conditions at labor market entry on health outcomes in later life in Japan.

## INTRODUCTION

A growing amount of evidence has demonstrated the adverse impact of macroeconomic downturns on population health. Studies have shown that the Great Recession (2008–2009) associated with the global financial crisis had negative effects on mental health, mortality, self-rated health (SRH), and other health outcomes.^1^^–^^4^ The possible key channels that link macroeconomic conditions and individual health include employment and financial strain; higher chances of unemployment and reduced income may raise health risks, especially among socioeconomically disadvantaged individuals.^5^^–^^8^ Studies also observed the widening socioeconomic inequalities regarding health during the economic crisis, indicating that economic shocks have a stronger impact on socioeconomically disadvantaged individuals.^9^^–^^11^

Most preceding studies have compared population health during the pre- and post-crisis periods or focused on changes in population health during the crisis,^12^^,^^13^ leaving the lingering impact of the economic crisis largely understudied. Difficulties in finding good jobs during the recession upon graduating from college and high school may have long-lasting adverse impacts on health in later life. The impact of an unsuccessful start of working life is likely serious and lasting in a society that provides limited chances to move to better jobs.^14^^–^^16^ In such cases, comparing health outcomes of the cohort that started its working life during the economic crisis with those of other cohorts will provide new insights about the impact of macroeconomic conditions on population health.

The current study aims to examine the cohort-specific lingering impact of the recession in Japan, using repeated cross-sectional data. Japan experienced the “employment ice age” (EIA) during 1993–2004, when young people had difficulty in finding jobs after graduation and started their working lives under unfavorable wage conditions. The job openings-to-applicants ratio for those aged 20–24 years was less than one during this period,^17^ and the growth in average starting salary for new college graduates dropped sharply to about 0.4% per year during 1993–2004 from 4.2% during the previous 12 years.^18^ We focused on health outcomes of the cohort that entered labor force during the EIA. This cohort, referred to as the “EIA cohort” hereafter, was born during 1970–1985 based on year of graduation from college or high school. Studies on labor economics provided evidence that this cohort had more limited chances of becoming full-time, regular workers and obtained lower wages than other cohorts.^19^ Hence, it can be hypothesized that the EIA cohort faces poorer health outcomes in working life.

## MATERIALS AND METHODS

### Study sample

We used a large dataset obtained from the “Comprehensive Survey of Living Conditions” (CSLC), a nationwide population-based survey conducted by the Japanese government’s Ministry of Health, Labour and Welfare (MHLW). The CSLC, conducted since 1986, comprises an annual household survey and a triennial health and income/savings survey. Samples of the CSLC are collected nationwide through a two-stage random sampling procedure. First, about 5,400 districts are selected randomly from about 940,000 national census districts. Second, about 290,000 households are selected randomly from each selected district, according to its population size.

We used the data collected from each of eleven waves of the CSLC conducted from 1986 through 2016. We restricted the study sample to individuals aged between 30 years (when most individuals have already started working after graduating from college or high school) and 59 years (1 year prior to the most common mandatory retirement age of 60 years) to assess the impact of the experience of EIA on later working life. After excluding respondents for whom essential information was missing, this study used data on 3,054,782 individuals (1,500,618 men and 1,554,164 women). Table 1 presents the numbers of households and their members (individuals) who responded to each survey, the response rates at the household level, and the number of individual participants in this study.

**Table 1.** Numbers of respondents in the Comprehensive Survey of Living Conditions (CSLC) and of individual participants in this study

Wave	Survey year	Number of CSLC respondents (households)	Response rate for households (%)	Number of CSLC respondents (individuals)	Individuals used in this study		

					Total	Men	Women
1	1986	240,283	95.7	803,807	344,025	169,048	174,977
2	1989	250,609	93.1	803,228	339,061	167,073	171,988
3	1992	253,653	92.9	783,095	325,783	160,047	165,736
4	1995	247,229	91.0	746,592	305,708	150,258	155,450
5	1998	247,882	89.7	721,478	292,052	143,859	148,193
6	2001	247,278	87.4	703,399	283,842	139,734	144,108
7	2004	220,948	79.9	619,573	246,983	120,978	126,005
8	2007	230,596	80.1	624,171	249,800	122,409	127,391
9	2010	229,785	79.4	609,018	232,498	113,957	118,541
10	2013	235,012	79.6	603,211	226,596	110,800	115,796
11	2016	224,641	77.6	568,425	208,434	102,455	105,979

Total		2,627,916	—	7,585,997	3,054,782	1,500,618	1,554,164

This study was restricted to individuals aged 30–59 years for whom essential information was available.

We obtained the CSLC data with permission from the MHLW. The CSLC was authorized by the Ministry of Internal Affairs and Communications, which is in charge of all government surveys in Japan, from the statistical, legal, ethical, and other viewpoints in accordance with the Statistics Law in Japan. Hence, ethics approval was not required for the current study.

### EIA and the EIA cohort

**Figure 1.**
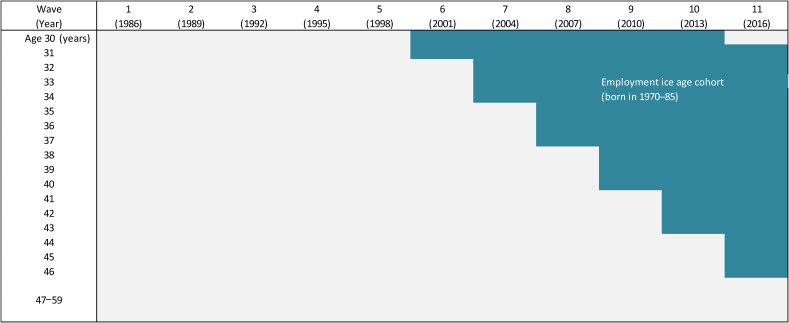
Appearance of the employment-ice-age cohort in the study sample

The EIA is defined as the period from 1993 through 2004 by the Cabinet Office of the Japanese Government.^20^ The EIA cohort, which started its working life in this period, was born during 1970–1985 in most cases, depending on the year of graduation from college or high school. The structure of the study sample is illustrated in Figure 1. The shadowed area indicates the appearance of the EIA cohort in the study sample. This cohort did not appear during waves 1–5 (1986–1998), but entered the study sample during wave 6 (2001), when individuals born in 1970 and 1971 were 31 and 30 years old, respectively. The number of individuals in the EIA cohort has been gradually increasing since then. By wave 11 (2016), the entire EIA cohort had entered the study sample, with members’ ages ranging from 31 to 46 years. In this wave, those aged 47 years or above (born before 1969) belonged to the pre-EIA cohort, and those aged 31 (born in 1986) were in the post-EIA cohort.

As seen in Figure 1, the EIA cohort was relatively young in the study sample and had been increasing its share in more recent waves. In the entire study sample, the EIA cohort consisted of 417,988 individuals, accounting for 13.7% of the total. In wave 11, the EIA cohort consisted of 109,467 individuals, accounting for 52.5% of the sample in this wave.

### Measures

#### Health outcomes

We considered three health outcomes—being in hospital, subjective symptoms, and SRH—all of which were obtained from responses in the CSLC. The CSLC asked the respondents whether they were in hospital during the survey period. We constructed a binary variable for being in hospital, allocating 1 to the respondents who answered yes and 0 otherwise. Those who were not hospitalized were questioned about the other two outcomes; in other words, those in hospital were excluded from the analysis of these outcomes. As for subjective symptoms, the survey asked, “Have you been feeling ill due to sickness or injury [subjective symptoms] for the past few days?” We constructed a binary variable for having any subjective symptoms, allocating 1 to those who answered yes to this question and 0 otherwise. Regarding SRH, the respondents were asked, “What is your current health status? Is it excellent, very good, good, fair, or poor?” We constructed two types of binary variables for SRH: (i) allocating 1 to *fair* or *poor* and 0 otherwise or (ii) allocating 1 to *poor* and 0 otherwise.

#### Covariates

We considered four covariates: having a spouse, having a paid job, household spending, and regional areas. For household spending, which was used as a proxy for household income, we first adjusted its reported value by the consumer price index (with the base year of 2015).^21^ Then, we adjusted it for household size by dividing it by the square root of the number of household members^22^ and categorized it into quartiles and binary variables of each quartile. We also constructed a binary variable of those who did not answer the question regarding household spending. For regional areas, we constructed binary variables for each of the eight areas (Hokkaido, Tohoku, Kanto, Chubu, Kinki, Chugoku, Shikoku, and Kyushu).

### Statistical analysis

We utilized two approaches to examine how health outcomes differed between the EIA cohort and other cohorts. First, we conducted the age-period-cohort (APC) analysis to capture the cohort effect.^23^ Specifically, we estimated logistic regression models to explain the binary variable of each health outcome, controlling for binary variables for each age and wave. The so-called identification problem, which is caused by the linear identity (age = period [wave] − cohort) and has been a key issue in the APC analysis,^24^^,^^25^ could be largely avoided in this study. This is because using repeated cross-sectional data, we considered binary variables, rather than continuous ones, for ages, waves, and cohorts with different year intervals—1 year for ages, 3 years for waves, and only two cohorts (EIA cohort vs other cohorts).^23^ We estimated two types of logistic models—with and without covariates (marital and work statuses, household spending, and regional areas)—for each health outcome, separately for male and female participants. Specifically, denoting the probability of each health outcome by *p*, we estimated the logistic regression model for individual *i*:

$$\begin{array}{rl} & \log(p_{i}/(1-p_{i}))=\text{constant}+\beta {\text{EIA cohort}}_{i}\\ & \;+\sum_{a=30}^{59}\gamma_{a}{\text{age}}_{ai}+\sum_{w=1}^{11}\delta_{w}{\text{wave}}_{wi}+({\text{covariates}}_{i})+\varepsilon_{i} \end{array}$$

where *EIA cohort*, *age*, and *wave* indicate the binary variables of the EIA cohort, each age, and each wave, respectively, and ε is an error term. Under the framework of mediation analysis,^26^^,^^27^ the difference in the estimated coefficient of the EIA cohort between the models with and without covariates is expected to reflect the EIA’s impact mediated by sociodemographic and socioeconomic factors.

**Figure 2.**
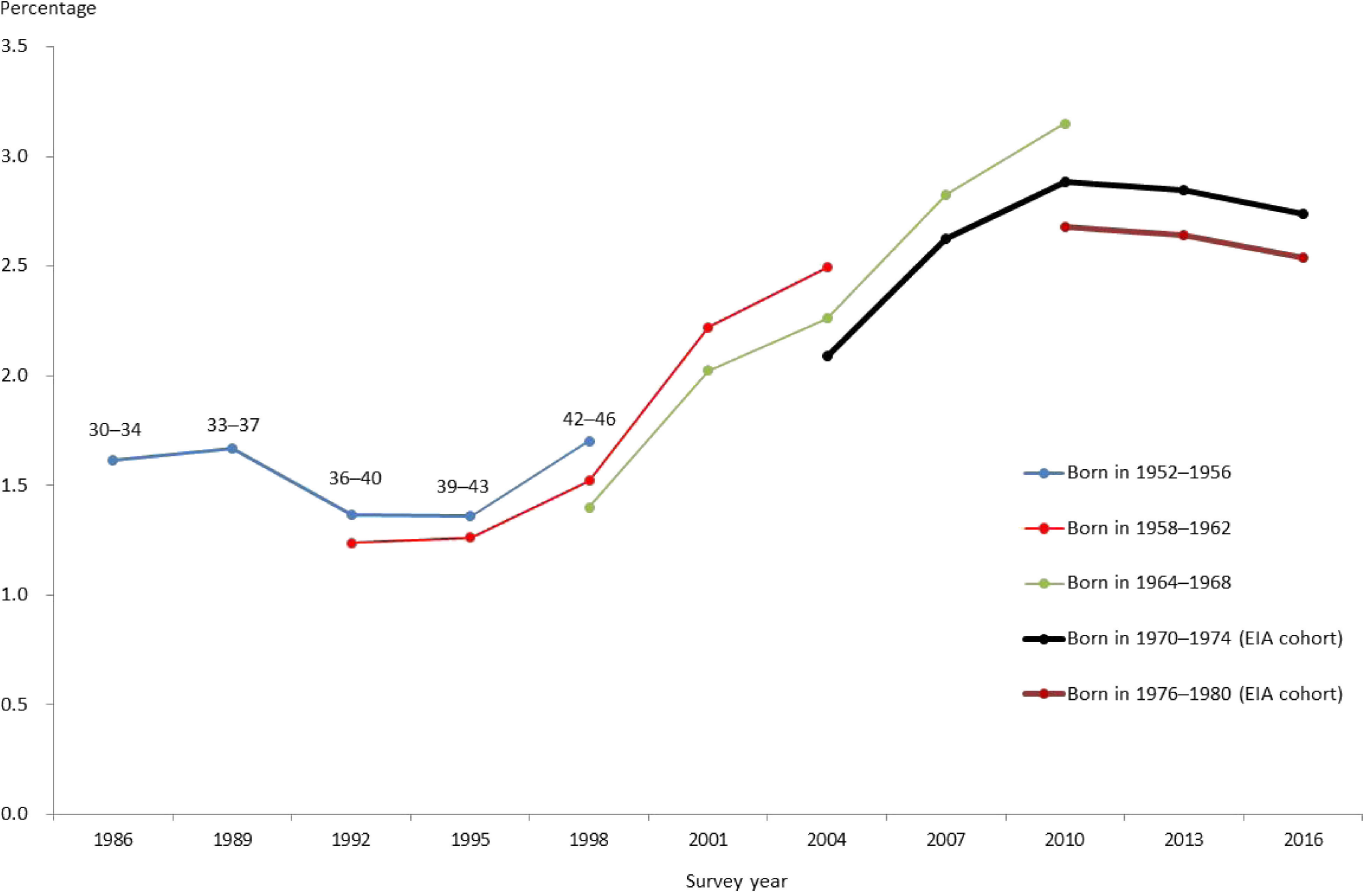
Age evolution of the prevalence of poor self-rated health by cohort: men. The dots in each curve indicate age (year) bands of 30–34, 33–37, 36–40, 39–43, and 42–46 years, respectively. Only the first three age bands for the cohort born in 1976–1980. EIA, employment ice age.

Second, we conducted a difference-in-differences (DiD) analysis.^28^^,^^29^ We compared changes in health outcomes during the pre- and post-EIA periods between the two age groups. Based on Figure 2, we chose waves 1–5 (ie, 1986–1998) and wave 11 (2016) as the pre-EIA and post-EIA periods, respectively. The EIA cohort did not appear in waves 1–5 at all, whereas it fully appeared in wave 11. As the two age groups, we chose individuals aged 31–46 years and others (ie, aged 30 or 47–59 years). Individuals aged 31–46 years supposedly faced the EIA if they appeared in wave 11 but did not if they appeared in waves 1–5. In contrast, individuals aged 30 or 47–59 years, regardless of whether or not they appeared in waves 1–5 or wave 11, are considered to have never faced such an experience. We thus treated those aged 31–46 years as the treatment group and others as the control group. We did not use the data of waves 6–10, in which the age group of 31–46 years included both the EIA and other cohorts, as seen in Figure 2.

To compare changes in health outcomes during the pre- to the post-EIA periods between two age groups, we estimated the logistic regression model to explain health outcomes by using (i) a binary variable of those aged 31–46 years, (ii) a binary variable of wave 11, and (iii) their interaction, with/without covariates separately for men and women. We also controlled for each age- and wave-specific factor by including binary variables for each age and wave in the regression models. Specifically, we estimated the following logistic regression model:

$$\begin{array}{rl} & \log(p_{i}/(1-p_{i}))=\text{constant}+\beta_{1}{\text{Aged 31–46 years}}_{i}\\ & \;+\beta_{2}\text{Wave}\;11+\beta_{3}{\text{Aged 31–46 years}}_{i}\times \text{Wave}\;11\\ & \;+\sum_{a=30}^{59}\gamma_{a}{\text{age}}_{ai}+\sum_{w=1}^{4}\delta_{w}{\text{wave}}_{wi}+({\text{covariates}}_{i})+\varepsilon_{i} \end{array}$$

where the second to forth terms on the right-hand side correspond to (i), (ii), and (iii), respectively, and ε is an error term. The estimated coefficient of the interaction term (β_3_) is expected to indicate the EIA’s impact. This type of regression model specification has been often used in DiD analysis, including in recent studies in Japan.^30^^,^^31^ To adjust for the potential bias due to the difference in age contributions between the pre-EIA (wave 1–5) and post-EIA (wave 11) periods, we weighted the data of each age in wave 11 with the ratio of the share of respondents of that age in wave 1–5 to the share of respondents of the same age in wave 11 in the DiD regression models.

This DiD approach assumes that if there is no EIA, health outcomes would change from the pre-EIA to the post-EIA period in parallel ways, resulting in a non-significant coefficient of the interaction term.^28^^,^^29^ To evaluate the validity of this equal trends assumption, we compared the prevalence trends of each health outcome between the control and treatment groups before the EIA. The DiD approach with cross-sectional data additionally assumes that whether an respondent is observed before or after the treatment is independent of his/her observed outcome, given that he/she belongs to the treatment or control group.^32^ This additional assumption clearly held in this study, because the treatment and control groups were distinguished solely by age in each wave.

## RESULTS

### Descriptive analysis

Table 2 summarizes key features of all the respondents in this study. Note that the figures in the table are sample means not adjusted for ages. The propositions of those having a spouse and a paid job and belonging to higher quartiles of household spending are somewhat lower for the EIA cohort for both genders, probably reflecting the younger ages of its members. The prevalence of each poorer health outcome was somewhat lower for the EIA cohort than for other cohorts, again possibly reflecting the younger ages of its members. The prevalence of subjective symptom was somewhat higher among women, but other health outcomes did not show a substantial gender difference.

**Table 2.** Key features of the study sample

		Men			Women		
	
		EIA cohort	Other cohorts	Total	EIA cohort	Other cohorts	Total
Age, years	*M*	36.0	46.2	44.8	36.0	46.2	44.8
	*SD*	(4.3)	(8.1)	(8.5)	(4.3)	(8.2)	(8.5)
Proposition, %							
Having a spouse		60.6	82.5	79.5	68.1	83.8	81.7
Having a paid job		91.5	94.6	94.1	52.3	55.9	55.4
Household spending							
1st quartile (lowest)		25.5	20.0	20.8	26.6	19.7	20.6
2nd quartile		23.3	22.8	22.9	23.5	22.6	22.8
3rd quartile		23.8	24.2	24.2	24.0	24.4	24.3
4th quartile		20.7	25.9	25.2	19.2	26.4	25.4
Not answered		6.8	7.0	7.0	6.8	6.9	6.9
Health outcome							
Being in hospital		1.0	1.1	1.1	0.9	0.9	0.9
Any subjective symptom		26.4	26.9	26.8	28.8	32.2	31.8
Self-rated health (poor or fair)		10.0	10.5	10.4	9.3	11.7	11.4
Self-rated health (poor)		1.2	1.1	1.1	0.9	1.0	1.0

*N*		205,374	1,295,244	1,500,618	212,614	1,341,550	1,554,164

EIA, employment ice age.

Figures other than age were not adjusted for age.

**Figure 3.**
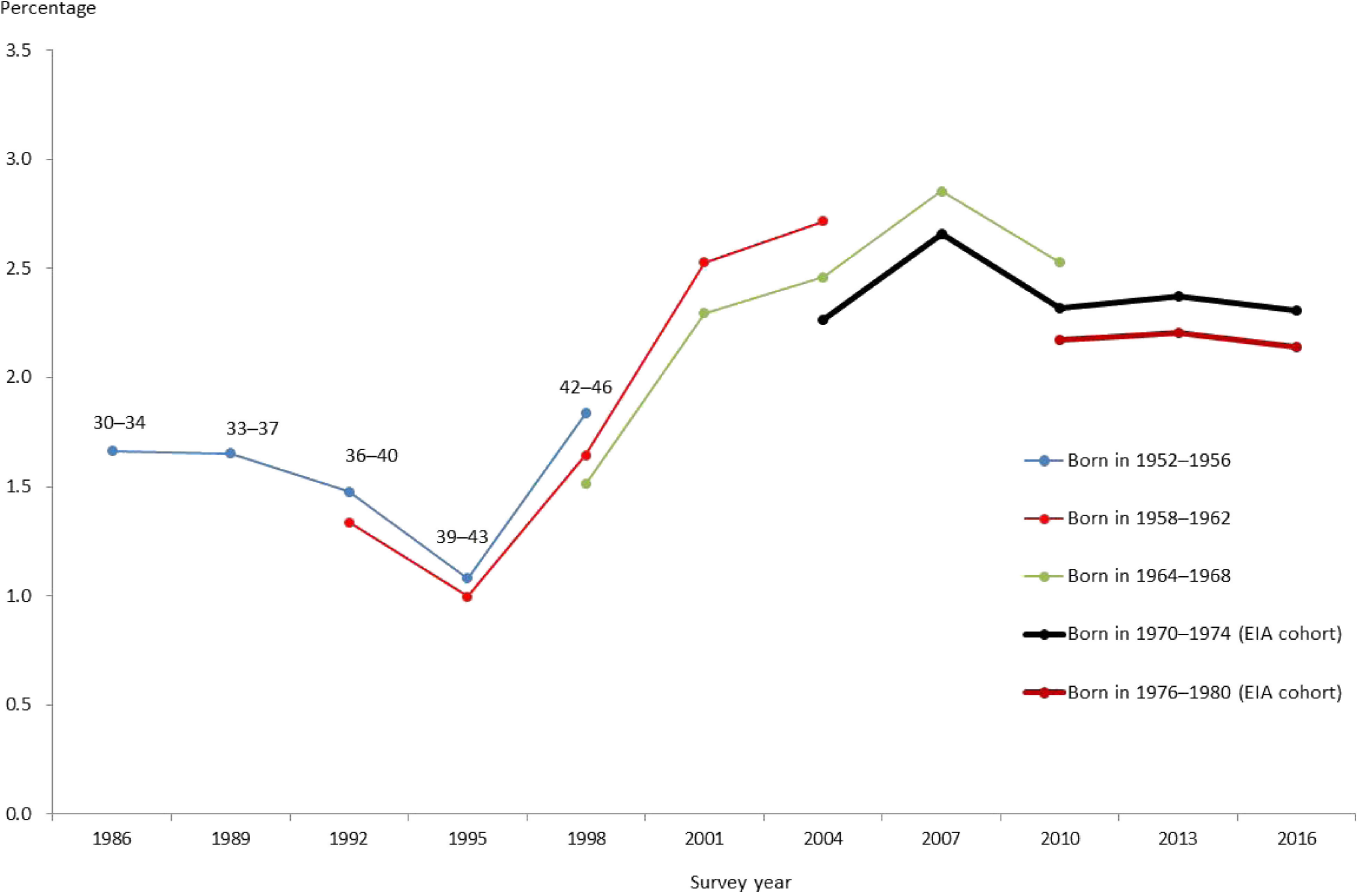
Age evolution of the prevalence of poor self-rated health by cohort: women. The dots in each curve indicate age (year) bands of 30–34, 33–37, 36–40, 39–43, and 42–46 years, respectively. Only the first three age bands for the cohort born in 1976–1980. EIA, employment ice age.

**Figure 4.**
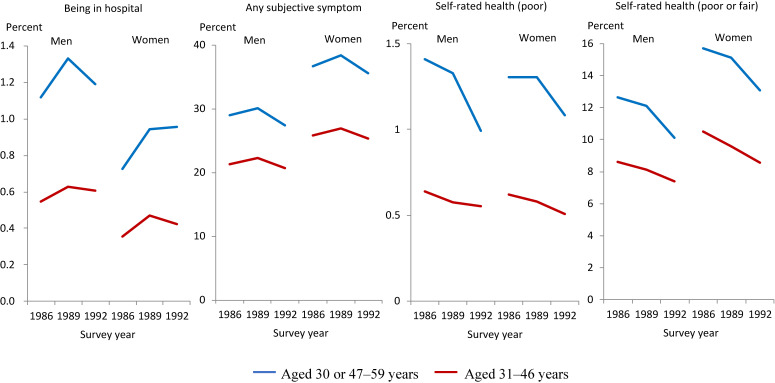
Prevalence trends of “any subjective symptom” and “poor self-rated health” before the employment ice age

Figure 3 and Figure 4 illustratively compare the age evolution of the prevalence of poor SRH across five cohorts for men and women, respectively, focusing on the age range of 30–46 years (30–40 years for the cohort born in 1976–80). The two youngest cohorts were born during 1970–74 and 1976–80, respectively, and started their working life during the EIA. The three older cohorts, who were born during 1952–1956, 1958–1962, and 1964–1968, started their working life before the EIA.

Figure 3 for men shows that the prevalence of poor SRH has been rising after bottoming out in the early 1990s through variations across cohorts, probably reflecting the changes in macroeconomic conditions; the trend of the unemployment rate turned from downward to upward, and the wage growth rate declined sharply in the early 1990s. By cohort, the prevalence of poor SRH for the two EIA cohorts started at high levels and, since then, stayed relatively high compared with the previous three cohorts. Figure 4 demonstrates similar results for women, showing higher prevalence of poor SRH for the two EIA cohorts, and this prevalence showed a clearer upturn for women than for men in 1995.

### Regression analysis

Table 3 summarizes the results of the regression models in the APC analysis, focusing on the estimated odds ratios (ORs) of each health outcome for the EIA cohort after controlling for ages and waves. The table compares the results with and without controlling for covariates. Based on the ORs, we found that the EIA cohort faced poorer health outcomes compared with other cohorts, for both men and women, regardless of whether or not covariates were controlled for. For instance, the OR of poor SRH for the male EIA cohort was 1.29 (95% confidence interval [CI], 1.21–1.38) without control for covariates and 1.25 (95% CI, 1.17–1.33) with control for covariates, while it was 1.25 (95% CI, 1.17–1.34) and 1.16 (95% CI, 1.08–1.24), respectively, for women. The ORs of poor/fair SRH were lower than those of other outcomes, but were significantly higher than one.

**Table 3.** Estimated odds ratios of each health outcome for the employment-ice-age (EIA) cohort, after controlling for age and wave effects

	Not controlling for covariates^a^		Controlling for covariates	

	OR	95% CI	OR	95% CI
Men (*N* = 1,500,618)				
Being in hospital	1.29	(1.21, 1.38)	1.29	(1.20, 1.37)
Any subjective symptom	1.20	(1.18, 1.21)	1.18	(1.17, 1.20)
Self-rated health (poor or fair)	1.06	(1.04, 1.09)	1.04	(1.02, 1.07)
Self-rated health (poor)	1.29	(1.21, 1.38)	1.25	(1.17, 1.33)

Women (*N* = 1,554,164)				
Being in hospital	1.19	(1.12, 1.28)	1.15	(1.08, 1.23)
Any subjective symptom	1.22	(1.21, 1.24)	1.21	(1.19, 1.22)
Self-rated health (poor or fair)	1.07	(1.04, 1.09)	1.02	(1.00, 1.05)
Self-rated health (poor)	1.25	(1.17, 1.34)	1.16	(1.08, 1.24)

CI, confidence interval; OR, odds ratio.

^a^The covariates include having a spouse and paid job, household spending, and regional areas.

Another noticeable finding here is that the ORs were not much attenuated after controlling for covariates. Although not reported to conserve space, the regression analysis found that the EIA cohort had lower chances of having a spouse and paid job and higher household spending, all of which were negatively associated with poorer health outcomes. These results point to the possibility that sociodemographic and socioeconomic factors mediated the impact of EIA experience on health in later life. However, the results in Table 3 suggest that such mediating effects, if any, were relatively limited, especially among men.

We should be cautious in interpreting the results in Table 3 because the EIA cohort may have worse health outcomes due to some events that affected the individuals before they started their working lives. To assess this potential bias, Table 4 compares each health outcome during school age (16–22 years) between the EIA and other cohorts for both men and women. Out of eight gender and health outcome combinations, three yielded worse outcomes and three better for the EIA cohort, while the outcomes of two combinations were not significantly different between the EIA and other cohorts. Table 3 presents the estimated EIA effects on being in hospital for men and on any subjective symptom for both genders; these effects are relatively substantial, but are probably somewhat overestimated.

**Table 4.** Comparison of school-age (16–22 years) health outcomes between the employment-ice-age (EIA) and other cohorts^a^

Health outcome prevalence (%)	EIA cohort	Other cohort	Difference	*P*-value
	(A)	(B)	(A − B)	
Men (*N* = 239,519)				
Being in hospital	0.52	0.40	0.12	<0.001
Any subjective symptom	15.04	14.23	0.80	<0.001
Self-rated health (poor or fair)	4.21	5.37	−1.16	<0.001
Self-rated health (poor)	0.40	0.52	−0.12	0.003

Women (*N* = 234,816)				
Being in hospital	0.41	0.36	0.05	0.114
Any subjective symptom	18.99	18.39	0.60	0.002
Self-rated health (poor or fair)	5.27	6.88	−1.61	<0.001
Self-rated health (poor)	0.39	0.42	−0.03	0.348

^a^The data relate to respondents born during 1926–1986.

Table 5 shows key results of the DiD analysis, presenting the estimated ORs of binary variables of individuals aged 31–46 years, wave 11, and their interaction, with and without controlling for covariates. The ORs of individuals aged 31–46 years were all well below one in all models, a reasonable result considering that those individuals were relatively young. The ORs of wave 11 were mixed, suggesting that the direction of wave effects depended on the type of health outcomes. More importantly, ORs of the interaction between individuals aged 31–46 years and wave 11 were all above one for all health outcomes for both men and women, before controlling for covariates. This result indicated that the EIA cohort had poorer health outcomes compared with the other cohorts. ORs of the interaction declined after controlling for covariates but remained above one for all health outcomes among men, while they declined to below one for being in hospital and poor/fair SRH among women.

**Table 5.** Relationship of health outcomes with “aged 31–46 years,” “wave 11,” and their interaction: a difference-in-differences analysis^a^

	Aged 31–46 years^b^		Wave 11		Aged 31–46 years × Wave 11	
		
	OR	95% CI	OR	95% CI	OR	95% CI
Not controlling for covariates^c^						
Men (*N* = 892,740)						
Being in hospital	0.46	(0.40, 0.53)	1.15	(1.04, 1.27)	1.43	(1.26, 1.62)
Any subjective symptom	0.63	(0.61, 0.65)	1.02	(0.99, 1.04)	1.23	(1.19, 1.27)
Self-rated health (poor or fair)	0.63	(0.59, 0.66)	1.26	(1.22, 1.30)	1.07	(1.02, 1.11)
Self-rated health (poor)	0.49	(0.42, 0.58)	1.62	(1.48, 1.78)	1.39	(1.24, 1.56)
Women (*N* = 922,323)						
Being in hospital	0.53	(0.45, 0.62)	1.52	(1.37, 1.68)	1.16	(1.02, 1.32)
Any subjective symptom	0.62	(0.60, 0.64)	0.74	(0.72, 0.75)	1.28	(1.24, 1.31)
Self-rated health (poor or fair)	0.69	(0.66, 0.72)	0.90	(0.87, 0.93)	1.06	(1.01, 1.11)
Self-rated health (poor)	0.46	(0.39, 0.55)	1.27	(1.15, 1.41)	1.42	(1.24, 1.62)

Controlling for covariates						
Men (*N* = 892,740)						
Being in hospital	0.56	(0.48, 0.65)	0.94	(0.85, 1.04)	1.40	(1.24, 1.59)
Any subjective symptom	0.67	(0.64, 0.69)	1.01	(0.99, 1.03)	1.22	(1.18, 1.26)
Self-rated health (poor or fair)	0.70	(0.66, 0.73)	0.96	(0.92, 0.99)	1.05	(1.00, 1.09)
Self-rated health (poor)	0.66	(0.56, 0.77)	0.96	(0.87, 1.05)	1.33	(1.18, 1.49)
Women (*N* = 922,323)						
Being in hospital	0.65	(0.55, 0.77)	1.88	(1.69, 2.08)	1.06	(0.93, 1.21)
Any subjective symptom	0.64	(0.61, 0.66)	0.78	(0.76, 0.80)	1.26	(1.23, 1.30)
Self-rated health (poor or fair)	0.75	(0.71, 0.79)	0.72	(0.70, 0.74)	1.01	(0.97, 1.05)
Self-rated health (poor)	0.60	(0.51, 0.70)	0.95	(0.86, 1.04)	1.25	(1.09, 1.42)

CI, confidence interval; OR, odds ratio.

^a^The estimates are adjusted for differences in age distributions between those aged 31–46 years (treatment group) and others (control group).

^b^This age group supposedly faced the employment ice age at labor market entry only if they appeared in wave 11 (in 2016).

^c^The covariates include having a spouse and paid job, household spending, and regional areas.

Figure 4 compares the prevalence trends of each health outcome between the control and treatment groups before the EIA. The prevalence of each health outcome moved largely in tandem between the control and treatment groups in the pre-EIA waves, though to a greater or lesser degree depending on the gender and heath outcome combination, suggesting that the equal trends assumption largely held in this study sample.

## DISCUSSION

We examined whether the health outcomes differed between the cohorts who started working life during the recession and the other cohorts. Specifically, we focused on the cohort that experienced adverse macroeconomic conditions during 1994–2003 (the EIA) when they started their working life in Japan. The results in this study showed that starting working life during the recession had a long-lasting impact on health outcomes for both men and women. The APC analysis showed that the EIA cohort had poorer health outcomes, even after controlling for age and cohort effects. This observation was underscored by the DiD analysis, which treated the experience of EIA as a treatment implemented on those aged 31–46 years.

The results of the current study are in line with previous observations about the impact of economic recessions on population health,^5^^–^^8^ as well as the disadvantage of the EIA cohort in terms of job status and wage earnings in Japan.^20^ The results are also consistent with previous observations in Japan,^33^ which confirmed that starting working life with a precarious job status tends to result in lower SES and poorer health outcomes. This implies that these individuals have socio-institutional backgrounds that generally limit chances to move to better jobs after entering the labor market as precarious employees.^34^ The limited mediating effects of sociodemographic and socioeconomic factors observed in this study, especially among men, may reflect the general perception that failure at the start of one’s working life is difficult to overcome and presumably has a traumatic and lingering impact on health. The observations of the current study did not show any significant gender differences before controlling for covariates, but results of the DiD analysis after controlling for them suggested that the traumatic impact of adverse economic conditions at labor market entry is more relevant for men.

We should be cautious in making any generalizations based on the observations in this study. The impact of macroeconomic conditions at the start of one’s working life on health outcomes in later life must depend on socio-institutional backgrounds. The impact is likely to be more mixed in societies with more flexible job markets, wherein there are sufficient chances to find better jobs, even if the economic downturn generally increases financial strain and health risks, as suggested by preceding studies in European countries.^14^^–^^16^

We acknowledge that this study has several limitations, in addition to concerns about the reliability of self-reported health outcomes and ignorance of intra-cohort variations. Our statistical analysis relied on repeated cross-sectional data, which made it impossible to track the impact of the recession experience on health outcomes in later life at an individual level. We controlled for age and wave cohorts by including binary variables for each of them in the APC analysis but ignored the interaction effects among ages, waves, and cohorts. We argued for limited mediating effects of sociodemographic and socioeconomic factors, but experiencing an economic downturn may have other mediators for its impact on health outcomes.

Despite these limitations, this study highlighted the lingering impact of starting working life during a recession on health outcomes in Japan. This finding, though dependent on socio-institutional backgrounds, provides new insights about the association between macroeconomic conditions and population health. It also implies the need for structural reforms in the labor market from the viewpoint of public health in Japan.
